# Role of concomitant endomyocardial biopsy in non-ischemic cardiomyopathy with ventricular arrhythmias

**DOI:** 10.1007/s10840-025-02221-6

**Published:** 2026-01-23

**Authors:** Daisuke Togashi, Yumi Katsume, Shunsuke Uetake, Salah H. Alahwany, William G. Stevenson, Harikrishna S. Tandri, Arvindh N. Kanagasundram, Travis D. Richardson

**Affiliations:** Department of Medicine, Cardiovascular Division, Vanderbilt University Medical Center, Vanderbilt Heart and Vascular Institute, 1215 21 st Ave South, MCE 5th Floor, South Tower, Nashville, TN 37232 USA

**Keywords:** Endomyocardial biopsy, Ventricular tachycardia, Premature ventricular contraction, Non-ischemic cardiomyopathy

## Abstract

**Background:**

In patients with non-ischemic cardiomyopathy (NICM) presenting with ventricular arrhythmias (VAs), the diagnostic yield and clinical significance of routine endomyocardial biopsy (EMB) remain unclear. This study aimed to evaluate the diagnostic utility of EMB with electrophysiological procedures in patients with NICM.

**Methods:**

We enrolled 37 consecutive patients with NICM complicated by VAs who underwent EMB during electrophysiological procedures. Clinical history, imaging data, and genetic testing results were collected, and changes in diagnosis based on EMB findings were assessed.

**Results:**

The median age was 61 years, and 35 of 37 patients (94.6%) were male. The most common arrhythmia was sustained VT in 27 patients (73.0%), followed by frequent PVCs in 6 (16.2%) and non-sustained VT in 4 (10.8%). Twenty-nine patients (78.4%) underwent catheter ablation, while eight (21.6%) underwent device implantation. EMB identified cardiac amyloidosis in 3 patients (8.1%) via Congo red staining, while the remaining 34 (91.9%) showed nonspecific fibrosis. No complications occurred during EMB. Among the 11 patients (29.7%) who underwent positron emission tomography/computed tomography (PET-CT), six (54.5%) were diagnosed with cardiac sarcoidosis. Genetic testing was performed in 23 patients (62.2%), revealing pathogenic TTN mutations in 2 (8.7%) and an RYR2 mutation (4.3%) consistent with catecholaminergic polymorphic VT. Cardiac MRI was performed in 29 patients (78.4%), with late gadolinium enhancement observed in 2 patients (6.9%) with suspected myocarditis, 1 (3.4%) with suspected sarcoidosis, and 2 (6.9%) with dilated cardiomyopathy. Furthermore, 2 patients (6.9%) showed right ventricular dysfunction with wall motion abnormalities, consistent with arrhythmogenic right ventricular cardiomyopathy.

**Conclusion:**

EMB can be safely performed during electrophysiological procedures and may aid diagnosis in selected cases, particularly cardiac amyloidosis. However, PET-CT demonstrated greater overall diagnostic utility.

**Graphical Abstract:**

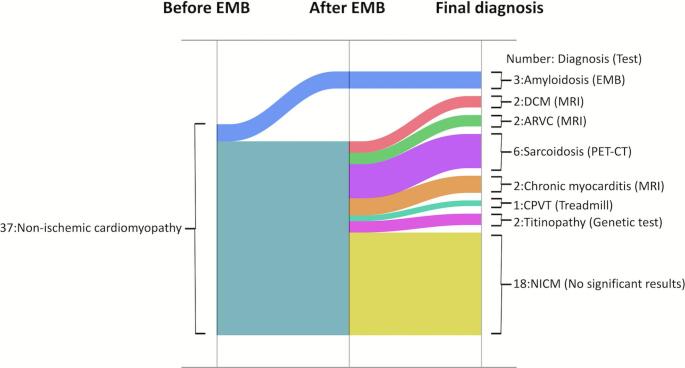

## Introduction

In patients with cardiomyopathy and ventricular arrhythmias (VAs), such as premature ventricular contractions or ventricular tachycardia (VT), identifying the etiology of cardiomyopathy plays a key role in determining treatment strategies, including radiofrequency catheter ablation (RFCA) approaches. Endomyocardial biopsy (EMB) is a valuable diagnostic tool in selected cases of non-ischemic cardiomyopathy (NICM) [1, 2], but its diagnostic yield varies depending on the suspected type of cardiomyopathy.

Recently, several reports have described improvements in diagnostic yield by performing EMB guided by electroanatomic voltage mapping (EAM) [3, 4]. However, it remains unclear to what extent routine EMB performed concomitantly with electrophysiologic procedures contributes to the diagnosis of underlying disease in patients with newly identified VAs and NICM. We investigated the role of routine EMB performed at the time of RFCA or cardiac implantable electronic devices (CIED) implantation.

## Methods

### Patient population

The decision to pursue EMB was made at the discretion of the treating electrophysiologist and was limited to patients without a definitive diagnosis at the time of evaluation.

This study included consecutive patients with NICM and VAs who underwent EMB as part of their clinical evaluation, along with concomitant ablation or CIED implantation, at Vanderbilt University Medical Center between January 2021 and March 2025. The study was approved by an Ethics Review Board in accordance with the Declaration of Helsinki. EMB was performed in line with current guidelines set forth by the American Heart Association, the American College of Cardiology, and the European Society of Cardiology [2]. The phenotypes of the various cardiomyopathies were determined with reference to the ESC guidelines [5]. Further investigation was performed using multiple modalities at the discretion of the treating physician, including magnetic resonance imaging (MRI), and positron emission tomography/computed tomography (PET-CT).

### Electroanatomic map

EAM was performed in a subset of patients who underwent catheter ablation. Mapping was performed using the CARTO™ 3D system (Biosense Webster, Inc., Irvine, CA, USA), incorporating intracardiac echocardiography and a Decanav multipolar mapping catheter. RFCA was performed using a ThermoCool SmartTouch or Surround Flow catheter (Biosense Webster, Inc.). Low-voltage areas (LVAs) were defined as regions with a bipolar voltage < 1.5 mV and/or unipolar voltage < 5.5 mV within the RV, as previously described [3]. Successful ablation sites were defined as those where VT was terminated and became non-inducible after ablation, or where PVCs were eliminated.

### Endomyocardial biopsy

EMB samples from the RV were obtained via the right femoral vein using a disposable bioptome (Bipal, Cordis, Miami, FL) introduced into a steerable sheath (Agilis NxT, St Jude Medical) or VIZIGO (Biosense Webster, Inc, Irvine, CA, USA). During CIED implantation, a 9Fr sheath was advanced into the axillary vein prior to device placement, and endomyocardial biopsy samples were obtained using a bioptome. In some cases, the bioptome was introduced through a curved sheath with a septal bias (e.g., SSPC2, Boston Scientific). Myocardial sampling was performed with intracardiac echocardiography and fluoroscopic guidance. When available, EAM was used to guide the bioptome to LVAs (both bipolar and unipolar) on the RV septum. No samples were obtained from regions outside of the RV septum. Samples were fixed in formalin and embedded in paraffin, followed by histological analysis, immunohistochemistry or immunofluorescence, and electron microscopy evaluation.

### Outcome measures

The outcome measures included changes in diagnosis before and after EMB and the incidence of procedure-related complications. In addition, heart failure–related hospitalization and all-cause mortality were assessed. Follow-up information was obtained from medical records, and the duration of follow-up was defined as the period from the index procedure to the last clinical contact or death.

### Statistics

Continuous variables are presented as mean ± standard deviation values or median (interquartile range [IQR]) and were compared using Student’s t-test or Wilcoxon’s rank sum test, depending on the distribution of the variables. Categorical values were also presented as counts and percentages. Statistical significance was considered achieved at *P* < 0.05, and analysis was performed using JMP® 18 (SAS Institute, Cary, NC, USA).

## Results

### Patient demographics

Of the 37 patients, the mean age was 61.4 years, and 94.6% were male (Table 1). Hypertension was present in 26patients (70.3%), and diabetes mellitus in 13 patients (35.1%). Eleven patients (29.7%) were NYHA class Ⅲ or Ⅳ. The median left ventricular ejection fraction was 37.5%. Atrial fibrillation was present in 11 patients (29.7%). At baseline, the right branch block was present in 13 patients (35.1%) and the left bundle branch block in 8 patients (22.2%). A detailed summary of the patients is shown in Table 2. Sustained VT (73.0%) was the most common diagnosis, followed by non-sustained VT (10.8%) and premature ventricular contractions (16.2%). Of the 37 patients, 29 underwent RFCA, with 25 receiving RV EAM. In the remaining eight patients, EMB was performed concomitantly with CIED implantation(including five implantable cardioverter defibrillators, two pacemakers, and one cardiac resynchronization therapy defibrillator). Based on echocardiographic phenotyping, 22 patients (59.5%) were diagnosed with non-dilated left ventricular cardiomyopathy, and 10 patients (27.0%) had left ventricular dilation.

**Table 1:** Baseline characteristics

Characteristics	EMB *n* = 37
Age, years	61.4 ± 14.2
Male, *n*(%)	35 (94.6)
Hypertension, *n*(%)	26 (70.3)
Diabetes, *n*(%)	13 (35.1)
Smoking, *n*(%)	17 (47.2)
NYHA Ⅲ or Ⅳ, *n*(%)	11 (29.7)
Type of VA	
Sustained VT, *n*(%)	27 (73.0)
NSVT *n*(%)	4 (10.8)
PVC *n*(%)	6 (16.2)
LVEF, %	37.5 [17.0–55.2]
RVFAC, %	37.6 [17.4–41.8]
TAPSE, mm	18.4 ± 7.6
Phenotype pattern by echocardiography*	
NDLVC	22 (59.5)
DCM	10 (27.0)
RCM	1 (2.7)
HCM	2 (5.4)
RVC	2 (5.4)
Restrictive cardiography	
Atrial fibrillation, *n*(%)	11 (29.7)
SSS, *n*(%)	2 (5.4)
RBBB, *n*(%)	13 (35.1)
LBBB, *n*(%)	8 (22.2)
AVB, *n*(%)	6 (16.2)
1 st degree, *n*(%)	3 (8.1)
3rd degree, *n*(%)	3 (8.1)

Categorical variables are presented as *n*(%)

Continuous variables are displayed as the mean ± standard deviation or median [Q1–Q3]

*The Phenotype was classified according to echocardiographic observations, which does not represent a definitive diagnosis

*AVB* atrioventricular block, *DCM* dilated cardiomyopathy, *EMB* endomyocardial biopsy, *LBBB* left bundle branch block, *LVEF* left ventricular ejection fraction, *NDLVC* non-dilated left ventricular cardiomyopathy, *NSVT* non-sustained ventricular tachycardia, *PVC * premature ventricular contraction, *RBBB* right bundle branch block, *RCM* restrictive cardiomyopathy, *RVC* right ventricular cardiomyopathy, *RVFAC* right ventricular fractional area change, *SSS* Sick sinus syndrome, *TAPSE* tricuspid annular plane systolic excursion, *VA* ventricular arrhythmia, *VT* ventricular tachycardia

**Table 2:** Patient summary

No	Age	Disease	Procedure	Other tests			Kind of VA	RV LVA on BV		RV LVA on UV		Biopsy Results	Clinical outcomes			
													F/U (days)	HF		Death
				MRI	PET	Genetic		cm^2^	%	cm^2^	%					
1	59	NICM	ABL	Yes	No	Yes	VT	25.8	13.6	41.9	22.1	Fibrosis, hypertrophy	1415	No		No
2	66	NICM	ABL	Yes	No	Yes	NSVT	3.7	2.5	33.7	22.8	NS	659	Yes		No
3	56	NICM	ABL	Yes	Yes	Yes	VT	2.2	2.2	3.0	3.0	Fibrosis, hypertrophy	1581	Yes		No
4	81	NICM	ABL	Yes	No	Yes	VT	3.2	2.7	9.8	8.3	Fibrosis, hypertrophy	900	No		No
5	44	NICM	ABL	Yes	No	Yes	VT	3.2	2.6	5.8	4.7	Fibrosis, hypertrophy	126	No		No
6	71	NICM	ABL	No	No	No	PVC	6.8	3.8	7.3	4.1	NS	265	No		No
7	67	NICM	ABL	No	Yes	Yes	VT	8.5	5.1	18.2	10.9	NS	695	No		No
8	68	NICM	ABL	Yes	No	Yes	VT	2.9	2.5	25.0	21.6	NS	154	No		No
9	55	NICM	ABL	Yes	No	Yes	VT	2.1	1.2	12.0	6.8	NS	573	Yes		Yes
10	61	NICM	ABL	Yes	No	No	PVC	3.2	1.5	15.0	7.0	NS	384	No		No
11	66	NICM	ICD	Yes	Yes	Ye	AVB, NSVT	N/A	N/A	N/A	N/A	NS	717	No		Yes
12	66	NICM	ABL	Yes	No	Yes	VT	N/A	N/A	N/A	N/A	NS	214	No		Yes
13	26	NICM	ABL	Yes	No	Yes	VT	N/A	N/A	N/A	N/A	NS	642	No		Yes
14	44	NICM	ABL	No	No	Yes	VT	N/A	N/A	N/A	N/A	NS	158	No		No
15	67	NICM	ABL	Yes	No	Yes	VT	25.3	21.1	35.4	29.5	Fibrosis, hypertrophy	350	No		No
16	78	NICM	ABL	Yes	No	Yes	VT	32.2	22.6	40.0	28.2	Fibrosis, hypertrophy	53	No		No
17	78	NICM	ABL	Yes	No	Yes	VT	16.0	8.9	22.2	12.3	Fibrosis, hypertrophy	1095	Yes		No
18	46	NICM	ABL	Yes	No	No	VT	12.5	8.9	23.5	16.8	Fibrosis, hypertrophy	135	No		No
19	55	NICM	ABL	Yes	No	No	VT	35	29.2	28	23.3	Fibrosis, hypertrophy	176	No	No	
20	44	NICM	ABL	Yes	No	Yes	VT	N/A	N/A	N/A	N/A	Fibrosis, hypertrophy	154	No	No	
21	62	ARVC	ABL	Yes	Yes	Yes	VT	15.6	7.5	5.2	2.5	Fibrosis, hypertrophy	196	No	No	
22	47	ARVC	ABL	Yes	Yes	Yes	VT	15.8	12.5	54.4	29.8	NS	1854	No	No	
23	77	Sarcoidosis	ABL	Yes	Yes	No	PVC	5.8	3.5	12.5	7.5	NS	1082	Yes	No	
24	70	Sarcoidosis	ABL	Yes	Yes	No	VT	1.5	1.2	21	16.8	Focal fibrosis	110	No	No	
25	64	Sarcoidosis	ABL	Yes	Yes	Yes	VT	3.7	2.1	5.6	3.1	NS	1482	Yes	No	
26	79	Sarcoidosis	ABL	No	Yes	No	PVC	100.0	72.0	75.4	54.5	NS	121	No	No	
27	74	Sarcoidosis	ICD	Yes	Yes	No	AVB, NSVT	N/A	N/A	N/A	N/A	NS	1303	No	No	
28	61	Sarcoidosis	ABL	No	Yes	No	VT	72.6	48.4	77.2	51.5	Fibrosis, hypertrophy	212	No	No	
29	78	Titinopathy	ABL	Yes	No	Yes	VT	4.6	2.30	12.1	6.2	Fibrosis, hypertrophy	152	No	No	
30	48	Titinopathy	ABL	Yes	No	Yes	VT	2.1	1.2	2.2	1.3	Fibrosis, hypertrophy	1022	No	No	
31	57	Chronic myocarditis	CRTD	Yes	No	No	VT	N/A	N/A	N/A	N/A	NS	1502	No	No	
32	50	Chronic myocarditis	ICD	Yes	No	Yes	VT	N/A	N/A	N/A	N/A	NS	1154	No	No	
33	71	Chronic myocarditis	ICD	No	No	Yes	VT	N/A	N/A	N/A	N/A	NS	71	No	No	
34	72	ATTR-AML	PM	Yes	No	No	NSVT	N/A	N/A	N/A	N/A	Congo-red positive	72	No	No	
35	75	ALκ- AML	PM	No	No	Yes	SSS, PVC	N/A	N/A	N/A	N/A	Congo-red positive	75	No	No	
36	67	ATTR-AML	ICD	No	No	Yes	AVB, PVC	N/A	N/A	N/A	N/A	Congo-red positive	694	Yes	No	
37	22	CPVT	ABL	Yes	No	Yes	NSVT	10.5	5.9	11	6.2	NS	1380	No	No	

*ABL* catheter ablation, *ARVC* arrhythmogenic cardiomyopathy, *AML* amyloidosis, *AVB* atrioventricular block, *CPVT* catecholaminergic polymorphic ventricular tachycardia, *CRTD* cardiac resynchronization therapy defibrillator, *EAM* electroanatomic map, *HF* heat failure, *ICD* implantable cardioverter-defibrillator, *NS n*on-specific, *LVA* low voltage area, *NICM* non-ischemic cardiomyopathy, *NS* non-specific, *NSVT* non-sustained ventricular tachycardia, *PM* pacemaker, *PVC* premature ventricular contraction, *RV* right ventricle, *RVOT* right ventricular outflow tract, *SSS* sick sinus syndrome, *VT* ventricular tachycardia

### Mapping data

**Fig. 1:**
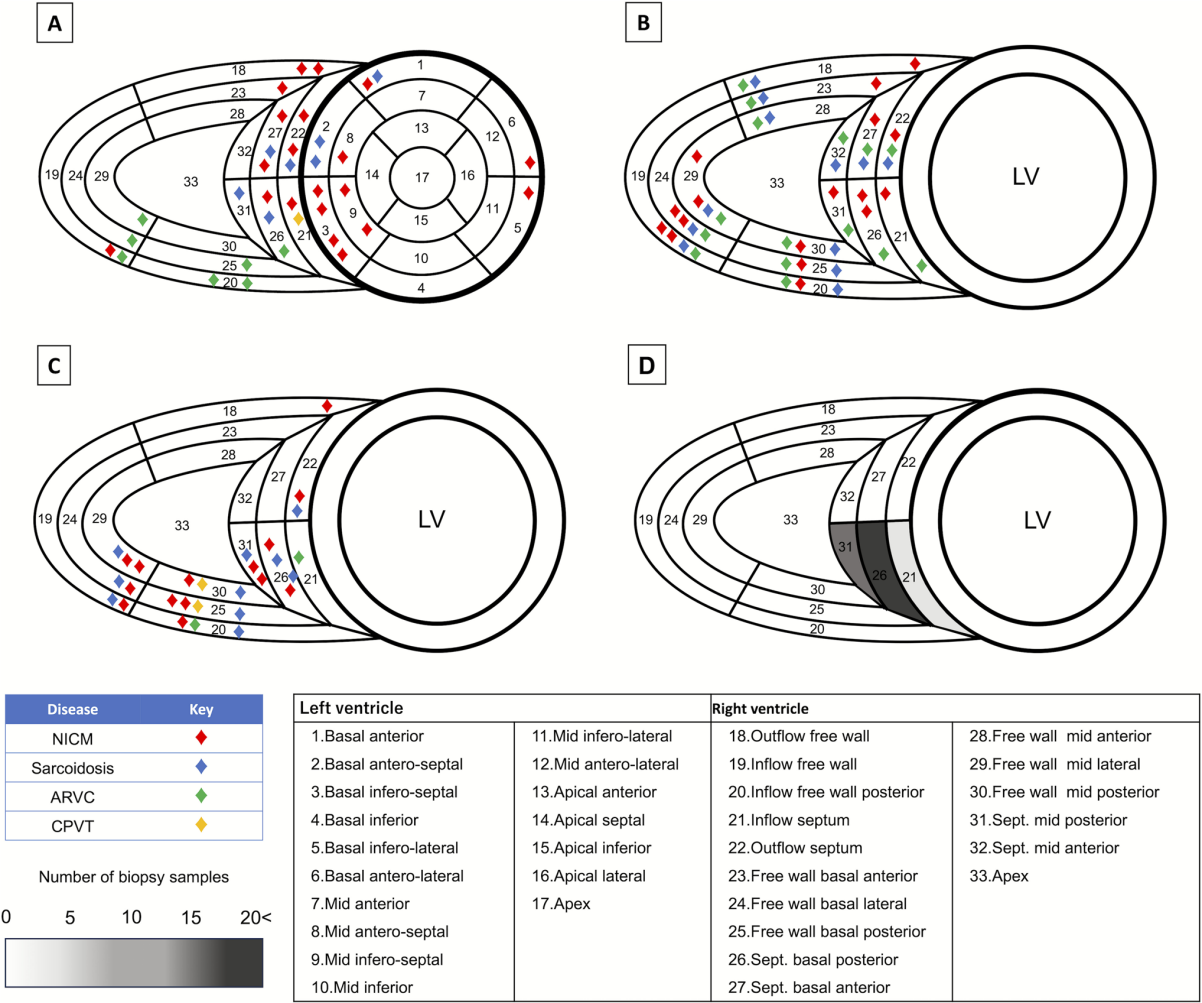
Distribution of EAM and EMB. Successful points for ventricular arrhythmia suppression (**A**), the localization of LVA identified on unipolar voltage mapping (< 5.5 mV) (**B**) and bipolar voltage mapping (< 1.5 mV) (**C**), and endomyocardial biopsy sites in the RV (**D**) are presented. On unipolar voltage mapping, LVAs were diffusely distributed across all patient groups. Successful ablation sites of ventricular arrhythmias were predominantly located in the right ventricular free wall and inferior inflow region in ARVC, the interventricular septum in Sarcoidosis, and extensive regions spanning both ventricles in NICM. EMB was most commonly performed in the septal region. ARVC, arrhythmogenic right ventricular cardiomyopathy; CPVT, catecholaminergic polymorphic ventricular tachycardia; EAM, electroanatomic voltage map; EMB, endomyocardial biopsy; LV, left ventricle; LVA, low voltage area; NICM, non-ischemic cardiomyopathy; RV, right ventricle

**Fig. 2:**
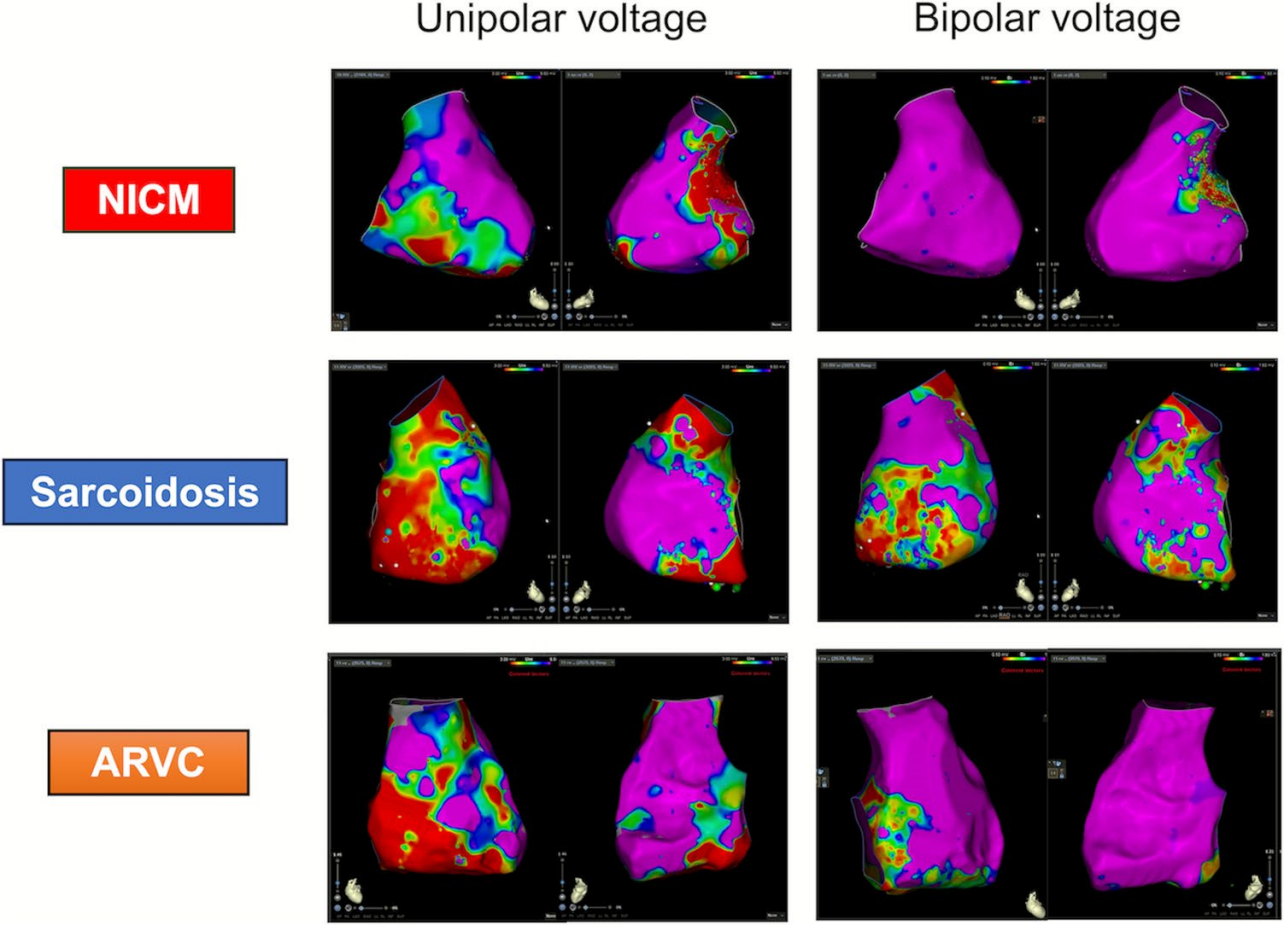
Typical examples of electroanatomic voltage maps. Top panel: NICM, middle panel: sarcoidosis, bottom panel: ARVC. In NICM, LVA at the basal inferior wall is observed only on a unipolar voltage map. In sarcoidosis, LVA on unipolar voltage overlaps with those observed on bipolar voltage. In ARVC, LVA at the basal lateral wall is identified only on unipolar voltage. Abbreviations are as in Fig. 1

The total procedure time, including EMB, was 276 [IQR 179–421] min. RV EAM was successfully obtained in 25 patients (Table 3). The VAs were mapped to the left ventricle in 14 patients, RV in 10 patients, and biventricular in 5 patients. Unipolar LVA was significantly larger than bipolar LVA (9.3 cm^2^ [IQR 2.3–35.0] vs. 3.5 cm^2^ [IQR 1.7–13.3], *P* = 0.006). In patients with sarcoidosis, bipolar LVA was noted in the RV septum and inferior wall. In arrhythmogenic right ventricular cardiomyopathy (ARVC), bipolar LVA was only seen in the RV inflow (Fig. 1A and B). Successful sites for suppression of VA were located in the RV free wall and inferior inflow region for ARVC, the interventricular septum for sarcoidosis, and multiple areas across both ventricles for patients with undifferentiated NICM (Fig. 1C). A typical EAM for each cardiomyopathy is shown in Fig. 2.

**Table 3:** Procedural data

Data	EMB *n* = 37
Procedure time, min	276 [179–421]
Numbers of samples, *n*	4.3 ± 1.2
Device implantation, *n *(%)	8(21.6)
PM, *n *(%)	2(5.4)
ICD, *n *(%)	5(13.5)
CRTD, *n *(%)	1(2.7)
Successful ablation site of VA	
LV, *n *(%)	14(37.8)
RV, *n *(%)	10(27.0)
Bi-ventricle, *n *(%)	5(13.5)
Points of EAM, *n*	1935 [1488–2399]
RV area, cm^2^	180.9[152–205.9]
RV Unipolar LVA, cm^2^	9.3[2.3–35.0]
RV Unipolar LVA, %	6.1[1.5–23.2]
RV Bipolar LVA, cm^2^	3.5[1.7–13.3]
RV Bipolar LVA, %	5.2[2.6–22.9]

Categorical variables are presented as *n *(%)

Continuous variables are displayed as the mean ± standard deviation or median [Q1–Q3]

*EAM* electroanatomic map, *EMB* endomyocardial biopsy, *ICD* implantable cardioverter-defibrillator, *LV* left ventricle, *PM* pacemaker, *PVC* premature ventricular contraction, *RV* right ventricle, *VA* ventricular arrhythmia

### Endomyocardial biopsy and pathological finding

**Fig. 3:**
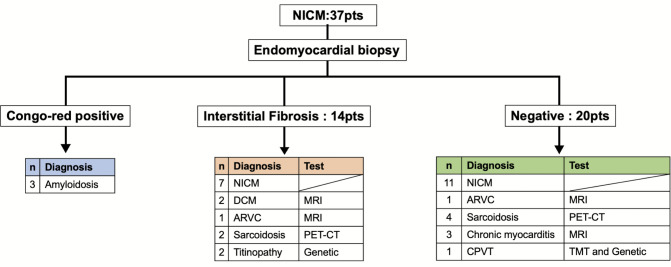
Flowchart for definitive diagnosis of non-ischemic cardiomyopathy. Among the NICM cases, three patients were diagnosed with amyloidosis based on endomyocardial biopsy findings. Of the 14 patients with interstitial fibrosis identified on EMB, two were diagnosed with DCM, one with ARVC based on cardiac MRI findings, and two with titinopathy confirmed by pathogenic TTN mutations. Additionally, two patients were diagnosed with sarcoidosis based on PET-CT findings. Among the remaining 20 patients with nonspecific or negative biopsy results, PET-CT identified four additional cases of cardiac sarcoidosis, while cardiac MRI revealed two cases of chronic myocarditis. Furthermore, one patient was diagnosed with ARVC based on MRI findings, and another was diagnosed with CPVT based on a positive RYR2 mutation and treadmill testing. ARVC, arrhythmogenic right ventricular cardiomyopathy; CPVT, catecholaminergic polymorphic ventricular tachycardia; DCM, dilated cardiomyopathy; MRI, magnetic resonance image; NICM, non-ischemic cardiomyopathy; PET-CT, positron emission tomography/computed tomography

The mean number of biopsy samples obtained was 4.3 ± 1.2 (Table 3). Most biopsies were taken from the basal to mid RV (Fig. 1D). At the biopsy sites corresponding to the septum, LVAs were identified in 10 patients on bipolar voltage mapping and in 12 patients on unipolar voltage mapping. A total of 51 samples (36.4%) were obtained from within the LVA region of the RV septum. Furthermore, late gadolinium enhancement (LGE) within the septal region was observed in two patients with dilated cardiomyopathy, two with chronic myocarditis, and one with sarcoidosis. Histologic abnormalities were identified in 17 of 37 (45.9%) cases (Fig. 3). Interstitial fibrosis was the most common finding, observed in 13 cases—each associated with EAM-guided biopsy. Of these, two patients were later diagnosed with titinopathy and one with ARVC. Congo-red staining was positive in two patients with hypertrophic cardiomyopathy pattern and one restrictive cardiomyopathy pattern on echocardiography, confirming amyloidosis (AL type in one, wild-type ATTR in the second). One patient had focal fibrosis and was later diagnosed with cardiac sarcoidosis. No granulomas were found in any biopsy specimens.

### PET-CT

PET-CT was performed in 11 patients. In 6 patients (54.5%), FDG uptake was noted in the basal inferoseptal region, consistent with cardiac sarcoidosis. Among the six patients suspected of having sarcoidosis, the diagnostic yield of EMB was 0%, although focal fibrosis was seen in one case. Three of the six underwent EAM-guided biopsy.

### MRI

Cardiac MRI was performed in 29 of 37 patients (78.4%). Subepicardial LGE in the basal septum was observed in two patients. One case showed LGE in the basal-lateral and basal-septal regions, consistent with sarcoidosis (also positive on PET-CT). Two patients showed reduced RV contractility and regional wall motion abnormalities, leading to a diagnosis of ARVC. Additionally, mid-septal LGE, typical of dilated cardiomyopathy, was seen in two patients.

### Genetic testing

Among the 23 cases (62.2%) that underwent genetic testing, pathologic mutations were identified in three patients, yielding a diagnostic rate of 13.0%. Two patients were found to have pathogenic TTN mutations, while one patient was identified with an RYR2 mutation and a positive treadmill test, leading to a diagnosis of catecholaminergic polymorphic VT. 

**Fig. 4:**
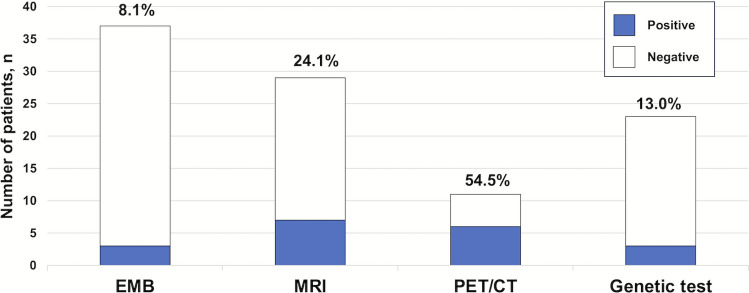
Diagnostic rate by each test. Of the patients undergoing each diagnostic test, a definitive diagnosis was achieved in 8.1% of cases using EMB, 24.1% using cardiac MRI, 54.5% using PET-CT, and 13.0% using genetic testing. Abbreviations are as in Figs. 1 and 3

The overall diagnostic yields for each evaluation modality were as follows: EMB in 3 of 37 cases (8.1%), cardiac MRI in 7 of 29 cases (24.1%), PET-CT in 6 of 11 cases (54.5%), and genetic testing in 3 of 23 cases (13.0%) (Fig. 4).

### Complications and clinical outcomes

No significant complications were observed related to EMB*.* There was no atrioventricular block related to EMB. In one case of epicardial VT ablation, a minor vessel injury occurred, leading to an increase in pericardial effusion, which required surgical vascular repair. This was not related to the EMB procedure. During a median follow-up of 385 days [153–1089], heart failure hospitalization occurred in 7 patients (18.9%), and death in 4 patients (10.8%) (Table 2).

## Discussion

**Fig. 5:**
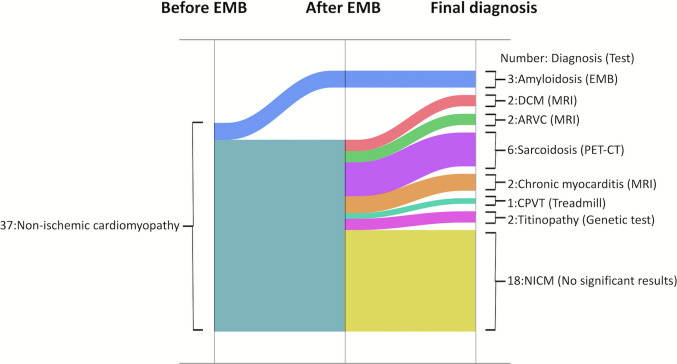
Sankey diagram of diagnoses before and after biopsy and at final diagnosis. After biopsy, only amyloidosis was confirmed, and no other specific diagnoses were made. Abbreviations are listed are as in Figs. 1 and 3

This study investigated how routine EMB contributes to diagnostic changes in patients with unexplained NICM complicated by VAs when procedures such as CIED implantation or RFCA are required. The main findings of this study are as follows: (1) only the diagnosis of amyloidosis was able to be confirmed by EMB alone (Fig. 5); (2) as previously reported, the diagnostic yield was low in sarcoidosis (0 of 6), PET-CT provided a definitive diagnosis in 6 cases, but no characteristic findings were observed on EMB; and (3) EMB most frequently identified interstitial fibrosis, which does not point to a specific diagnosis but may spur further investigation.

### EMB and other modalities

In patients with NICM, the annual risk of life-threatening VAs is approximately 1–2% [6]. Identifying the underlying etiology is essential for risk stratification and management. Current guidelines recommend EMB in patients presenting with unexplained VAs or bradyarrhythmias when an underlying cardiomyopathy is suspected [1, 2]. However, more recent expert consensus emphasizes that EMB should be performed selectively when a specific diagnosis is suspected that would directly influence management, and that routine use in patients with heart failure without such suspicion is not recommended [7]. In this context, EMB is particularly valuable in the diagnosis of infiltrative cardiomyopathies such as cardiac amyloidosis, in which diffuse myocardial deposition of misfolded proteins can be reliably detected histologically [8–10]. However, EMB has limited utility in cardiomyopathies with focal involvement, such as ARVC and cardiac sarcoidosis [2].

Advances in imaging have further restricted EMB’s role. Cardiac MRI allows comprehensive evaluation of ventricular function and tissue characterization through LGE and T1 mapping [11]. Specific fibrosis patterns may suggest particular etiologies and are linked to arrhythmia risk [12]. MRI is also central in evaluating suspected ARVC, detecting RV structural abnormalities and functional impairment [13]. However, MRI may not reliably differentiate ARVC from isolated RV sarcoidosis.

In cardiac sarcoidosis, PET-CT demonstrates higher sensitivity (89%) and moderate specificity (78%) for detecting active disease [14], and is therefore frequently used. By contrast, the diagnostic yield of EMB is modest, approximately 20% even with EAM guidance [15]. Some studies have reported higher yields (up to 40%) [16], possibly owing to sampling from non-septal regions. In our cohort, however, none of the patients with sarcoidosis had diagnostic histopathology despite EAM guidance, likely reflecting the limitation of septal-only sampling. Although no large studies have directly compared septal versus non-septal biopsy sites, prior data provide insight into this issue. Chimenti et al. reported that EMB performed from the RV apical septum was associated with tamponade and pericardial effusion in only 0.4% of cases [17]. In contrast, biopsies from the RV free wall have been considered higher risk, cautioning against such an approach due to an increased risk of tamponade [2, 17]. On the other hand, in selected diseases, non-septal sampling may increase diagnostic sensitivity. In ARVC, free wall biopsy has been reported to improve diagnostic yield because fatty replacement is most prominent in this region [13]. Left ventricular sampling carries the theoretical risk of severe embolic complications. These findings highlight the trade-off between safety and diagnostic sensitivity when considering biopsy location. Given the overall low yield of EMB for the majority of disease processes and the risks discussed, we feel non-septal targets should be avoided.

Genetic testing is another valuable tool in evaluating NICM. Its utility in identifying pathogenic mutations continues to grow, particularly in arrhythmogenic and dilated cardiomyopathies [18, 19]. Combining imaging and genetic data may enhance diagnostic accuracy where EMB is limited.

### Electroanatomic mapping and endomyocardial biopsy

EAM has been proposed to improve the diagnostic yield of EMB by targeting biopsy sites with abnormal voltage [3, 4, 9, 20, 21]. Notably, in our series, unipolar voltage abnormalities were more extensive than bipolar abnormalities, suggesting that the arrhythmogenic substrate may reside in intramural or epicardial layers, beyond the reach of standard biopsy tools. This is consistent with previous imaging studies showing intramural or epicardial fibrosis as a common finding in NICM [6, 11, 12].

Recent studies have refined our understanding of EMB’s role in NICM. Dello Russo et al. underscored the importance of a comprehensive workup, including imaging, electrophysiology, and biopsy, in athletes with VAs, suggesting that similar multifaceted approaches may benefit NICM patients [22]. Casella et al., in a study on lymphocytic myocarditis, demonstrated that EAM-guided EMB significantly increases diagnostic yield, particularly in inflammation-driven cardiomyopathy [23]. Another study by Casella et al. highlighted EMB’s underappreciated yet pivotal role in diagnosing arrhythmogenic cardiomyopathy and its inclusion in standardized ARVC criteria [24]. Taken together, these findings support the selective use of EMB, particularly when combined with EAM and advanced imaging, to enhance etiological and histopathological diagnosis in NICM with arrhythmias.

### Safety of EMB

No complications were observed attributable to EMB. The one complication in this study was clearly not due to the EMB but was attributable to the ablation procedure. Complications of EMB under conventional fluoroscopy have been reported to occur in approximately 6%, with cardiac perforation occurring in less than 1% [25]. However, it has been reported that the complication rate is lower under EAM, with no cardiac perforations observed [3, 4]. This is likely because using intracardiac echocardiography and EAM as a reference makes it possible to accurately visualize structures such as the RV and valves in real-time, ensuring safety.

## Limitations

This study has several limitations. First, it was a single-center, retrospective observational study with a relatively small sample size, which may limit the generalizability of the findings. Second, all biopsy specimens were obtained from the RV septum, and not all procedures were guided by EAM. Therefore, potential diagnostic findings in other myocardial regions may have been missed, even in cases with apparent bipolar voltage abnormalities. Third, the use and timing of additional diagnostic modalities—including CT, MRI, PET, and genetic testing—were not standardized or incorporated into a systematic diagnostic algorithm. A more structured approach might have reduced the diagnostic yield of EMB, as non-cardiac biopsy sites could have been selected for patients with suspected amyloidosis or sarcoidosis, thereby diminishing the need for EMB. Thus, the relatively low diagnostic yield observed in this study may still represent an overestimation.

## Conclusions

In patients with unexplained NICM presenting with VAs, EMB appears to be a safe procedure when performed using contemporary imaging and mapping guidance. However, the overall diagnostic yield of EMB remains limited, particularly when sampling is restricted to the RV septum. Routine performance of EMB at the time of ablation or CIED implantation may not be warranted in all patients with NICM and VA. Nonetheless, EMB may retain diagnostic value in selected cases, particularly when infiltrative or inflammatory cardiomyopathy is suspected.

## Data Availability

The data supporting the findings of this study are available from the corresponding author upon reasonable request.

## Abbreviations

ARVC: Arrhythmogenic right ventricular cardiomyopathy
CIED: Cardiac implantable electronic devices
EAM: Electroanatomic voltage map
EMB: Endomyocardial biopsy
LGE: Late gadolinium enhancement
LVA: Low voltage area
NICM: Non-ischemic cardiomyopathy
MRI: Magnetic resonance image
PET-CT: Positron emission tomography/computed tomography
RFCA: Radiofrequency catheter ablation
RV: Right ventricle or ventricular
VA: Ventricular arrhythmia
VT: Ventricular tachycardia
